# Hydrophilic Biocompatible Poly(Acrylic Acid-co-Maleic Acid) Polymer as a Surface-Coating Ligand of Ultrasmall Gd_2_O_3_ Nanoparticles to Obtain a High r_1_ Value and T_1_ MR Images

**DOI:** 10.3390/diagnostics11010002

**Published:** 2020-12-22

**Authors:** Yeong-Ji Jang, Shuwen Liu, Huan Yue, Ji Ae Park, Hyunsil Cha, Son Long Ho, Shanti Marasini, Adibehalsadat Ghazanfari, Mohammad Yaseen Ahmad, Xu Miao, Tirusew Tegafaw, Kwon-Seok Chae, Yongmin Chang, Gang Ho Lee

**Affiliations:** 1 Department of Chemistry, College of Natural Sciences, Kyungpook National University, Taegu 41566, Korea; yjijg@daum.net (Y.-J.J.); liushuwen0701@gmail.com (S.L.); 20100819@hanmail.net (H.Y.); sonlongh@gmail.com (S.L.H.); shantimarasini.sm@gmail.com (S.M.); adibeh.ghazanfari@gmail.com (A.G.); yaseen.knu@gmail.com (M.Y.A.); kitty0927@live.cn (X.M.); tegafawtirusew@yahoo.com (T.T.); 2 Division of Applied RI, Korea Institute of Radiological & Medical Sciences (KIRAMS), Seoul 01812, Korea; jpark@kirams.re.kr; 3 Department of Molecular Medicine, School of Medicine, Kyungpook National University, Taegu 41944, Korea; hscha1002@daum.net; 4 Department of Biology Education, Teachers’ College, Kyungpook National University, Taegu 41566, Korea; kschae@knu.ac.kr

**Keywords:** poly(acrylic acid-co-maleic acid), ultrasmall Gd_2_O_3_ nanoparticle, relaxivity, colloidal stability, biocompatibility, magnetic resonance imaging agent

## Abstract

The water proton spin relaxivity, colloidal stability, and biocompatibility of nanoparticle-based magnetic resonance imaging (MRI) contrast agents depend on the surface-coating ligands. Here, poly(acrylic acid-co-maleic acid) (PAAMA) (M_w_ = ~3000 amu) is explored as a surface-coating ligand of ultrasmall gadolinium oxide (Gd_2_O_3_) nanoparticles. Owing to the numerous carboxylic groups in PAAMA, which allow its strong conjugation with the nanoparticle surfaces and the attraction of abundant water molecules to the nanoparticles, the synthesized PAAMA-coated ultrasmall Gd_2_O_3_ nanoparticles (d_avg_ = 1.8 nm and a_avg_ = 9.0 nm) exhibit excellent colloidal stability, extremely low cellular toxicity, and a high longitudinal water proton spin relaxivity (r_1_) of 40.6 s^−1^mM^−1^ (r_2_/r_1_ = 1.56, where r_2_ = transverse water proton spin relaxivity), which is approximately 10 times higher than those of commercial molecular contrast agents. The effectiveness of PAAMA-coated ultrasmall Gd_2_O_3_ nanoparticles as a T_1_ MRI contrast agent is confirmed by the high positive contrast enhancements of the in vivo T_1_ MR images at the 3.0 T MR field.

## 1. Introduction

Magnetic resonance imaging (MRI) is a noninvasive imaging method that provides high-resolution three-dimensional images of the body [1]. Because it affords substantially good image contrasts for the brain and soft tissues, MRI is preferred for diagnosis of the brain and soft tissues. Further, contrast agents enhance the image resolution and sensitivity of MRI by increasing the contrast [2,3,4]. Hence, they can facilitate the early diagnosis of diseases.

The trivalent Gd^3+^, possessing the highest unpaired electron spin (S = 7/2) [5], is the most powerful element in the periodic table for accelerating the longitudinal (T_1_) water proton spin relaxation. Therefore, Gd^3+^-chelates are the most utilized T_1_ MRI contrast agents for clinical applications [2,3]. However, these chelates possess low longitudinal water proton relaxivity (r_1_) values (3 to 5 s^−1^mM^−1^) because they can only interact with one water molecule [2,3]. In contrast, gadolinium oxide (Gd_2_O_3_) nanoparticles with many Gd^3+^ on their surfaces can interact with many water molecules to provide much higher r_1_ values compared with the Gd-chelates [6,7,8,9]. Furthermore, as demonstrated by various types of ultrasmall nanoparticle systems [10,11,12], ultrasmall Gd_2_O_3_ nanoparticles can be excreted through the renal system similarly to molecular agents, which are essential for in vivo applications.

The good colloidal stability and biocompatibility of Gd_2_O_3_ nanoparticles are essential for their in vitro and in vivo applications. Moreover, better colloidal stability and biocompatibility can be obtained with larger ligands than with smaller ones [13], although the smaller ligands can achieve better renal excretion [10]. Further, the r_1_ values are affected by surface-coating ligands [14]: higher r_1_ values can be obtained by hydrophilic ligands than with hydrophobic ones [15,16]. In addition, the surface coating is required to prevent the release of free Gd^3+^ ions from the nanoparticles (free Gd^3+^ ions may cause nephrogenic systemic fibrosis if they are released in the body [17,18,19]). Therefore, the surface-coating of Gd-containing nanoparticles is necessary for in vitro and in vivo applications. For example, dextran and polyethylene-glycol-modified silica have been utilized for surface coating [20,21].

Here, poly(acrylic acid-co-maleic acid) (PAAMA) (M_w_ = ~3000 amu) was explored as a surface-coating ligand of ultrasmall Gd_2_O_3_ nanoparticles. It is known to be biocompatible [22], and comprises an almost equal number (~16) of AA and MA monomers. Each AA and MA monomer unit possesses one and two hydrophilic COOH groups, respectively. Many of them can be strongly conjugated to nanoparticle surfaces, thereby imparting the resulting nanoparticle colloids with stability in aqueous solutions. Notably, PAAMA was successfully used as a surface-coating ligand of iron oxide nanoparticles to provide good colloidal stability [23], and thus, applied for Gd_2_O_3_ nanoparticles herein. The performance of PAAMA as a surface-coating ligand was investigated by analyzing the synthesized PAAMA-coated ultrasmall Gd_2_O_3_ nanoparticles via various experimental techniques. Furthermore, the in vitro cellular toxicities and water proton spin relaxivities of the synthesized nanoparticles were measured, and their effectiveness as a potential T_1_ MRI contrast agent was investigated by obtaining in vivo T_1_ MR images of mice in a 3.0 T MR field.

## 2. Materials and Methods

### 2.1. Chemicals

GdCl_3_.*x*H_2_O (99.9%), triethyleneglycol (TEG, 99%), NaOH (>99.9%), and PAAMA (50 wt.% in water, M_w_ = ~3000 amu) were purchased from Sigma-Aldrich, St. Louis, MO, USA, and used as-received. Ethanol (99%) was purchased from Duksan Chemical Co., South Korea, and used as-received for the initial washing of the nanoparticles. Triple-distilled water was used for the final washing of the nanoparticles and preparation of the solution samples.

### 2.2. Synthesis of PAAMA-Coated Ultrasmall Gd_2_O_3_ Nanoparticles

**Figure 1:**
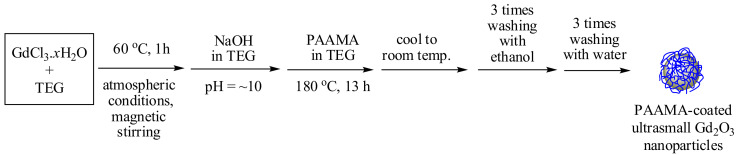
Reaction scheme (one-pot polyol synthesis) of the poly(acrylic acid-co-maleic acid) (PAAMA)-coated ultrasmall Gd_2_O_3_ nanoparticles. TEG = triethylene glycol.

The reaction scheme is shown in Figure 1. Three different solutions were prepared: (1) A total of 2 mmol of GdCl_3_.*x*H_2_O was dissolved in 20 mL of TEG in a three-necked round-bottom flask. The solution was stirred magnetically at 60 °C for 1 h under atmospheric conditions. (2) A total of 9 mmol of NaOH in 20 mL of TEG was prepared in a separate beaker, which was then added slowly to the abovementioned precursor solution until the pH of the solution reached ~10. (3) A total of 0.25 mmol of PAAMA prepared in 10 mL of TEG was added to the abovementioned solution. The mixture solution was magnetically stirred at 180 °C for 13 h. The product solution was cooled to room temperature, after which it was mixed with 400 mL of ethanol. The solution was magnetically stirred for 10 min and stored in a refrigerator until the product nanoparticles precipitated at the bottom of the beaker. The upper clear solution was decanted, and 400 mL of ethanol was added to the remaining solution. This washing process was repeated thrice. To remove ethanol from the product nanoparticles, the product solution was diluted with 400 mL of triple-distilled water and concentrated with a rotatory evaporator until the volume of the solution reached ~50 mL. This washing process was also repeated thrice. The concentrated solution was divided into two equal volumes: one half of the volume was diluted with triple-distilled water to produce a nanoparticle solution sample, and the other half of the volume was dried in air to prepare a powder sample for the various characterizations.

### 2.3. Characterizations

The particle diameter of the PAAMA-coated ultrasmall Gd_2_O_3_ nanoparticles was measured using a high-resolution transmission electron microscope (HRTEM) (Titan G2 ChemiSTEM CS Probe, FEI, Hillsboro, OR, USA) operated at 200 kV acceleration voltage. The Gd concentration of the aqueous solution sample was determined using an inductively coupled plasma atomic emission spectrometer (ICPAES) (IRIS/AP, Thermo Jarrell Ash Co., Waltham, MA, USA). The hydrodynamic diameter was measured using a dynamic light-scattering (DLS) particle size analyzer (Zetasizer Nano ZS, Malvern, Malvern, UK) and a 0.01 mM Gd solution sample. The zeta potentials (Zetasizer Nano ZS, Malvern, Malvern, UK) were measured using a nanoparticle suspension sample with 1.0 mM Gd. The crystal structure of the powder sample before and after thermogravimetric analysis (TGA) was measured using a multi-purpose X-ray diffraction (XRD) spectrometer (X’PERT PRO MRD, Philips, The Netherlands) with an unfiltered CuKα radiation (λ = 1.54184Å). A Fourier transform-infrared (FT-IR) absorption spectrometer (Galaxy 7020A, Mattson Instrument Inc., Madison, WI, USA) was used to investigate the surface coating of the nanoparticles using a powder sample pelletized with KBr. The amount of PAAMA coating on nanoparticle surfaces was estimated by recording the TGA curve (SDT-Q600, TA Instrument, New Castle, DE, USA) between room temperature and 900 °C while air flowed over the powder sample. A vibrating sample magnetometer (VSM) (7407-S, Lake Shore Cryotronics Inc., Westerville, OH, USA) was used to characterize the magnetic properties of the powder sample (20–30 mg) by recording the magnetization (M) versus applied field (H) (or M−H) curve (−2.0 T ≤ H ≤ 2.0 T) at 300 K. The net M value of the sample (only Gd_2_O_3_ nanoparticles without the PAAMA coating) was estimated using the net mass of the sample extracted from the TGA curve. 

### 2.4. In Vitro Cellular Toxicity Measurements

The in vitro cellular toxicity of the nanoparticle solution sample was assessed using a CellTiter-Glo Luminescent Cell Viability Assay (Promega, Madison, WI, USA). In this assay, a luminometer (Synergy HT, BioTek, Winooski, VT, USA) was used to quantify intracellular adenosine triphosphate. Two cell lines, such as human prostate cancer (DU145) and normal mouse hepatocyte (NCTC1469), were used as the test cells. Both cells were seeded on a 24-well cell culture plate and incubated for 24 h (5 × 10^4^ cell density, 500 μL of cells per well, 5% CO_2_, and 37 °C). Roswell Park Memorial Institute (RPMI) 1640 and Dulbecco’s Modified Eagle Medium (DMEM) were used as culture media for DU145 and NCTC1469 cells, respectively. Five test nanoparticle solutions were prepared by diluting the original concentrated nanoparticle solution sample (18.0 mM Gd) with a sterile phosphate-buffered saline solution, and 2 μL of each test nanoparticle solution sample was added to the above cultured cells to obtain Gd-concentrations of 10, 50, 100, 200, and 500 μM Gd in them. Thereafter, the treated cells with nanoparticle solutions were incubated for 48 h. A total of 200 μL of CellTiter-Glo reagent was added to 200 μL of the above incubated treated cells per well and the cells were lysed for 30 min on an orbital shaker. The viabilities of the cells were measured using the luminometer (300–700 nm), and normalized with respect to that of the control cells with 0.0 M Gd. The measurements were repeated in triplicate to estimate average cell viabilities.

### 2.5. Relaxometric Property Measurements

The longitudinal (T_1_) and transverse (T_2_) water proton spin relaxation times were measured at 22 °C using a 3.0 T MRI scanner (Magnetom Trio Tim, Siemens, Munchen, Bayern, Germany). A series of nanoparticle solution samples (0.5, 0.25, 0.125, and 0.0625 mM Gd) were prepared by diluting the original concentrated nanoparticle solution sample with triple-distilled water. Then, these dilute solutions were used for the measurements of the T_1_ and T_2_ relaxation times. The longitudinal (r_1_) and transverse (r_2_) water proton spin relaxivities were estimated from the slopes of the plots of 1/T_1_ and 1/T_2_ versus the Gd-concentration, respectively. The longitudinal (R_1_) and transverse (R_2_) relaxations were obtained from 1/T_1_ and 1/T_2_, respectively. R_1_ and R_2_ map images were then obtained from R_1_ and R_2_ values of the samples. The T_1_ relaxation times were measured by the inversion recovery method. In this method, the inversion time (TI) was varied at 3.0 T, and the MR images were acquired at 35 different TI values in the range from 50 to 1750 ms. The T_1_ relaxation times were then obtained from the nonlinear least-squares fits to the measured signal intensities at various TI values. For the multiple spin-echo measurements of the T_2_ relaxation time, the Carr–Purcell–Meiboon–Gill pulse sequence was employed. Thereafter, 34 images were acquired at 34 different echo time (TE) values in the range from 10 to 1900 ms. The T_2_ relaxation times were obtained from the nonlinear least-squares fits to the mean pixel values for multiple spin-echo measurements at various TE values. The following parameters were employed for the measurements: external MR field (H) = 3.0 T, temperature (T) = 22 °C, number of acquisitions (NEX) = 1, field of view (FOV) = 16 cm, FOV phase = 0.5, matrix size = 256 × 128, slice thickness = 5 mm, pixel spacing = 0.625 mm, pixel band width = 122.10 Hz, and repetition time (TR) = 2000 ms.

### 2.6. In Vivo T_1_ MR Image Measurements

The in vivo animal imaging experiments were performed according to the rules and regulation of the animal research committee of the Korea Institute of Radiological and Medical Sciences (approval number = Kirams2018-0072 and approval data: 2019-01-09). The same 3.0 T MRI scanner was used to obtain in vivo T_1_ MR images. Two male Balb/c nude mice (~20 g) were used. The mice were anesthetized using 1.5% isoflurane in oxygen. The measurements were performed before and after the injection of a nanoparticle solution sample into mice tail veins. The injection dose was approximately 0.1 mmol Gd/kg. During measurements, body temperature of the mice was maintained at 37 °C using a warm water blanket. After measurements, the mice were revived from anesthesia and placed in a cage with free access to food and water. For the in vivo images, radio-frequency spoiled T_1_-weighted, gradient-recalled echo (GRE) sequences were used. The typical measurement parameters are as follows: applied MR field (H) = 3.0 T, temperature = 37 °C, echo time = 12 ms, repetition time = 564 ms, pixel bandwidth = 15.63 Hz, frequency = 256 Hz, phase = 256, number of acquisitions = 3, field of view = 60 mm, phase field of view = 1, slice thickness = 1.0 mm, number of slices = 24, and spacing gap = 1.1 mm.

## 3. Results

### 3.1. Particle Diameter

**Figure 2:**
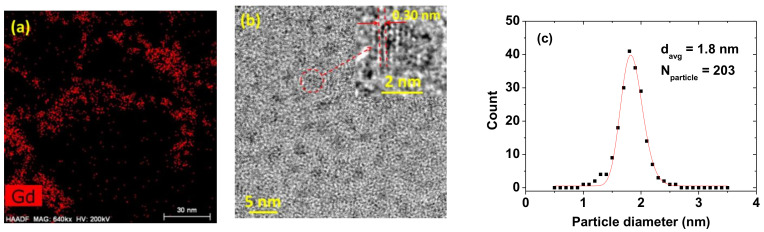
(**a**) High-angle annular dark field-scanning transmission electron microscope (HAADF-STEM) elemental mapping image exhibiting the dispersions of the PAAMA-coated ultrasmall Gd_2_O_3_ nanoparticles. (**b**) High-resolution transmission electron (HRTEM) image of the PAAMA-coated ultrasmall Gd_2_O_3_ nanoparticles [red dotted circle is magnified at the top (as indicated with an arrow) as an inset on a 2 nm scale with a (222) plane lattice distance (labeled as dotted lines and arrows) of 0.30 ± 0.01 nm]. (**c**) Particle diameter distribution and a log-normal function fit to obtain d_avg_ (N_particle_ is the total number of nanoparticles used for the fit).

The ultrasmall nanoparticle dispersions were identified through the Gd-elemental mapping of a high-angle annular dark field-scanning transmission electron microscope (HAADF-STEM) image on a 30 nm scale (Figure 2a). As shown in Figure 2a, the well-dispersed, PAAMA-coated ultrasmall Gd_2_O_3_ nanoparticles are observed. Thereafter, the particle diameters were estimated by measuring HRTEM images. As shown in Figure 2b, particle diameters are nearly monodispersed and ultrasmall, ranging from 1 to 3 nm. The inset is a magnified image of a nanoparticle (labeled as a dotted circle) on a 2 nm scale, which shows the lattice fringe distance of 0.30 ± 0.01 nm, matching that of (222) planes of cubic Gd_2_O_3_ [24]. The average particle diameter (d_avg_) was estimated to be 1.8 ± 0.1 nm using a log-normal function fit to the observed particle diameter distribution (Figure 2c and Table 1).

### 3.2. Colloidal Stability

**Figure 3:**
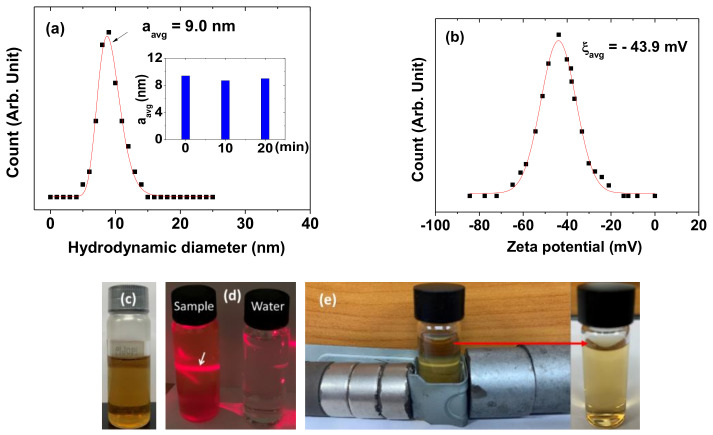
(**a**) Dynamic light-scattering (DLS) pattern and a log-normal function fit to obtain a_avg_. Inset is a plot of the a_avg_ measured as a function of time (min). (**b**) Zeta potential curve and a Gaussian function fit to obtain ξ_avg_. (**c**) Photograph of an aqueous nanoparticle solution sample showing the good colloidal dispersion without precipitation of PAAMA-coated ultrasmall Gd_2_O_3_ nanoparticles in solution. (**d**) The Tyndall effect (laser-light scattering) confirming the colloidal dispersion of PAAMA-coated ultrasmall Gd_2_O_3_ nanoparticles in solution. The effect (indicated with an arrow) was only observed in the nanoparticle solution sample (left vial); it was not observed in triple-distilled water (right vial). (**e**) Test for the colloidal stability in a magnetic field with no precipitation of the nanoparticles (experimental set-up (left) and a photograph of the solution sample after the experiment (right)).

The colloidal stability was investigated by measuring the hydrodynamic diameter and zeta potential, as well as through the inspection of any nanoparticle precipitation in an aqueous nanoparticle solution sample (18.0 mM Gd). The average hydrodynamic diameter (a_avg_) was estimated to be 9.0 ± 0.2 nm from the log-normal function fit of the observed DLS pattern (Figure 3a and Table 1). It was measured three times with time intervals of 10 min (inset in Figure 3a) and the values were consistent with each other. A large a_avg_ indicates that the PAAMA-coated ultrasmall Gd_2_O_3_ nanoparticles were extensively hydrated by many water molecules, thus explaining the observed good colloidal stability. This is attributable to the presence of many hydrophilic COO^-^ groups in the PAAMA polymers, which attracted many water molecules to the nanoparticles. Moreover, the high negative zeta potential (−43.9 mV) of the PAAMA-coated ultrasmall Gd_2_O_3_ nanoparticles in aqueous solutions also explains the observed good colloidal stability (Figure 3b). The aqueous nanoparticle solution sample did not show any precipitation of the PAAMA-coated ultrasmall Gd_2_O_3_ nanoparticles in the solution after synthesis (>1 year), further explaining the observed good colloidal stability (Figure 3c). The colloidal dispersion of the PAAMA-coated ultrasmall Gd_2_O_3_ nanoparticles in an aqueous solution was confirmed through laser-light scattering (the Tyndall effect, left vial in Figure 3d), which was not observed in the vial that contained triple-distilled water (right vial in Figure 3d). Because the COOH groups of PAAMA possess a much higher binding affinity to the nanoparticles than the OH groups of TEG, most of the TEG molecules were likely replaced with PAAMA during the surface-coating reaction for 13 h. Then, the synthesized nanoparticles were thoroughly washed with 400 mL of ethanol (three times) to remove the solvent (TEG) as much as possible, including other reactants. Hence, the TEG molecules, which remained in the sample, would be fewer or negligible. Notably, if most of the TEG were not removed from the nanoparticles, the colloidal stability would not be good because TEG-coated nanoparticles generally settle in a solution after a few days. In addition, the colloidal stability was tested in the presence of magnetic field employing strong neodymium permanent-magnet disks for seven days (Figure 3e). No precipitation of the nanoparticles was observed, thus confirming the good colloidal stability of the PAAMA-coated ultrasmall Gd_2_O_3_ nanoparticles in a magnetic field. The large hydrodynamic diameter might indicate the nanoparticle aggregation. However, because of the observed long-term good colloidal stability of the nanoparticle colloids (>1 year), the nanoparticle aggregation would not be severe even if it did exist.

### 3.3. Crystal Structure

**Figure 4:**
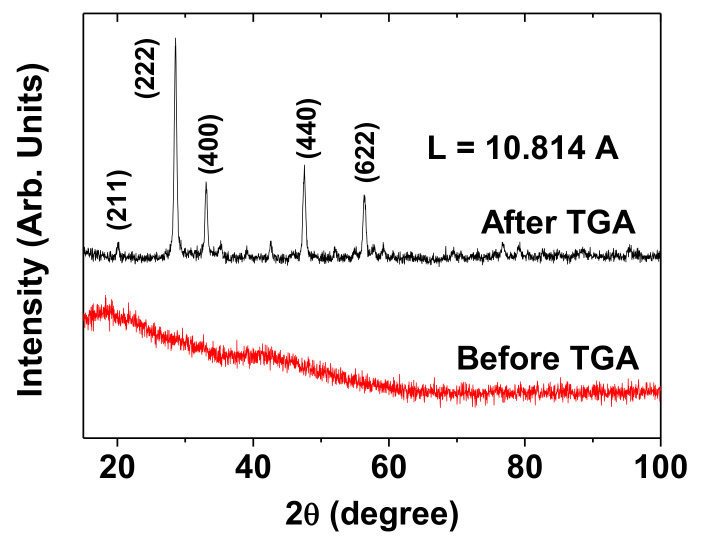
XRD patterns of the powder sample before (bottom spectrum) and after (top spectrum) thermogravimetric analysis (TGA). All the peaks after TGA could be assigned the (hkl) Miller indices of cubic Gd_2_O_3_, and only the intense peaks were representatively assigned in the XRD pattern. “L” = lattice constant.

The XRD patterns were recorded before and after TGA, as shown in the bottom and top XRD patterns in Figure 4, respectively. Before TGA, the XRD pattern was broad and amorphous because of the incomplete crystallization of the synthesized nanoparticles due to their ultrasmall particle sizes [25]. However, after TGA up to 900 °C, the XRD pattern exhibited sharp peaks, which corresponded to those of cubic Gd_2_O_3_ with the JCPDS card no. 43–1014 [24]. This could be attributed to the particle-size growth and crystallization of the nanoparticles after TGA [26]. All the peaks after TGA could be assigned the (hkl) Miller indices, and only the strong peaks were representatively assigned in the XRD pattern. The estimated lattice constant (L) after TGA was 10.814 Å, which was consistent with the previously reported value [24].

### 3.4. Surface-Coating Results

**Figure 5:**
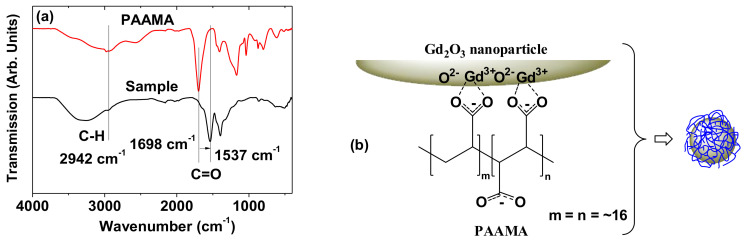
(**a**) FT-IR absorption spectra of the powder sample (bottom spectrum) and free PAAMA (top spectrum). The arrow indicates the red-shift of the C=O stretch. (**b**) Coating structure of PAAMA on the ultrasmall Gd_2_O_3_ nanoparticle surface. Each PAAMA was strongly bonded to the ultrasmall Gd_2_O_3_ nanoparticle surface through many coordination bonds between many COO^-^ groups of PAAMA and many Gd^3+^ on the nanoparticle surface (approximately six PAAMA polymers were coated per nanoparticle, as estimated from TGA).

The surface coating was investigated by recording the FT-IR absorption spectra and TGA curve. The C–H and C=O stretches of PAAMA were observed in the spectrum of the powder sample **(**Figure 5a), thus confirming the successful surface coating of the Gd_2_O_3_ nanoparticles with PAAMA. The coating structure of PAAMA on the nanoparticle surface is shown in Figure 5b. As shown in Figure 5b, many carboxylic groups in PAAMA strongly bonded to many Gd^3+^ on the nanoparticle surface. The COO^-^ stretch at 1537 cm^−1^ in the spectrum of the sample was red-shifted by 161 cm^−1^ from the C=O stretch at 1698 cm^−1^ of the free PAAMA, thereby confirming the strong coordination bonds. Such red-shifts, which have been observed in many surface-coated metal oxide nanoparticles with ligands containing carboxylic groups, further support our results [27,28,29,30]. This type of bonding corresponds to the hard acid (Gd^3+^ on the nanoparticle surface)−hard base (COO^-^ of PAAMA) type of bonding [31,32].

**Figure 6:**
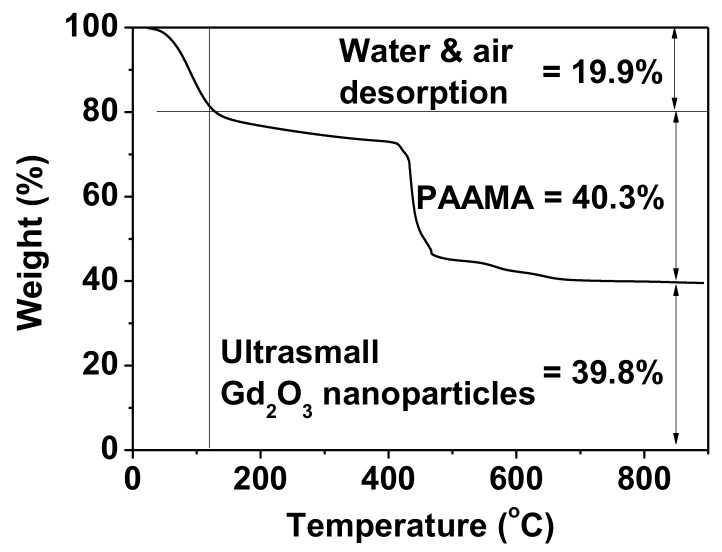
TGA curve of the powder sample exhibiting wt.% of PAAMA (40.3%) and that of the ultrasmall Gd_2_O_3_ nanoparticles (39.8%) after assessing wt.% of water and air desorption (19.9%) from the sample.

The amount (P) of PAAMA coating on the ultrasmall Gd_2_O_3_ nanoparticle surface was 40.3%, as estimated from the mass drop in the TGA curve that was caused by the PAAMA combustion reaction with flowing hot air after considering the initial mass drop of 19.9 wt.% between room temperature and ~105 °C due to water and air desorption (Figure 6). The remaining 39.8 wt.% was due to the Gd_2_O_3_ nanoparticles. Using the abovementioned P-value, d_avg_ from HRTEM imaging, and bulk density (7.41 g/cm^3^) of Gd_2_O_3_ [33], the grafting (or coating) density (σ) [34,35], which corresponds to the average number of PAAMA polymers coating a nanoparticle unit surface area, was estimated to be 0.48 nm^−2^. By multiplying σ by the nanoparticle surface area (=πd_avg_^2^), the average number (N) of PAAMA polymers coating a nanoparticle was estimated to be ~6. As each PAAMA (M_w_ = ~3000 amu), as a copolymer, comprises (AA)_m_ and (MA)_n_ monomer units (m = n = ~16) (Figure 5b), each PAAMA has ~48 COO^−^ groups because of the presence of one COO^-^ group per AA and two COO^-^ groups per MA. Thus, there are ~288 COO^-^ groups per nanoparticle (some of them are conjugated to the nanoparticle surface, while the others are free). The surface-coating results are summarized in Table 1.

### 3.5. Cell Viability

**Figure 7:**
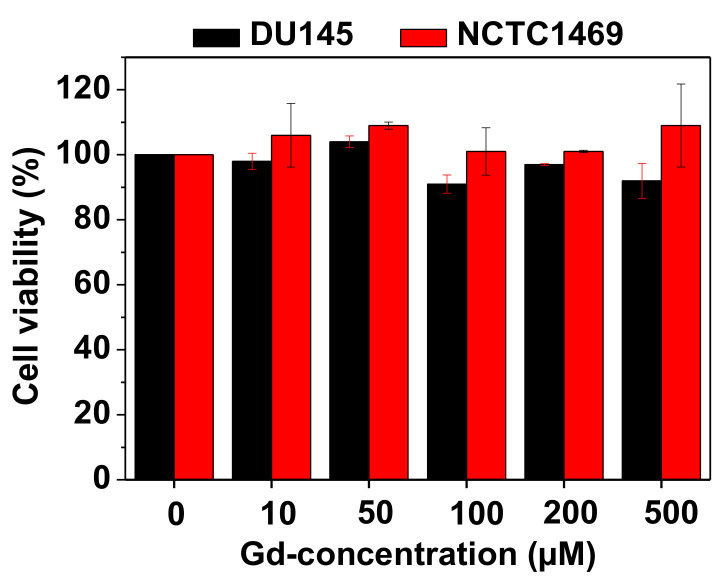
In vitro cell viabilities of the PAAMA-coated ultrasmall Gd_2_O_3_ nanoparticles on the DU145 and NCTC1469 cells as a function of Gd-concentration, which showed extremely low cellular toxicities.

The PAAMA-coated ultrasmall Gd_2_O_3_ nanoparticles were nearly non-toxic up to 500 μM Gd in the DU145 and NCTC1469 cell lines (Figure 7). This was due to the biocompatible PAAMA coating on the nanoparticle surfaces.

### 3.6. Magnetic Properties

**Figure 8:**
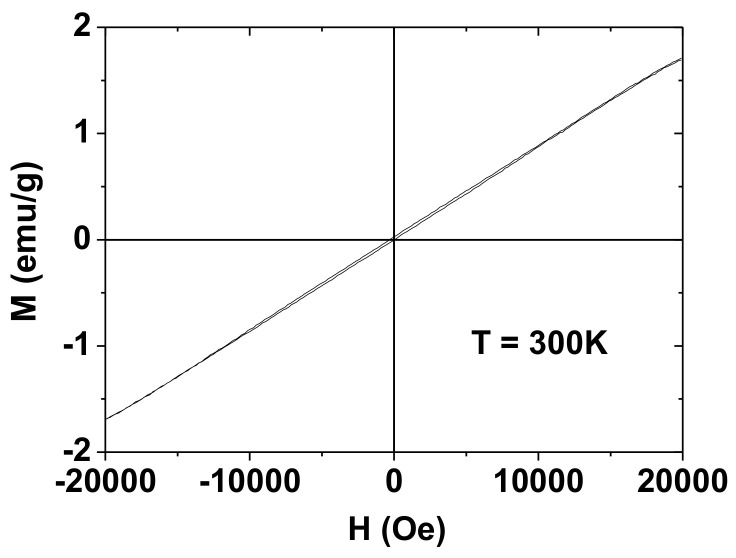
M-H curve of the PAAMA-coated ultrasmall Gd_2_O_3_ nanoparticles at 300 K, showing paramagnetism. The M value is the net M value of the ultrasmall Gd_2_O_3_ nanoparticles only (without PAAMA), which was estimated from the net mass of the ultrasmall Gd_2_O_3_ nanoparticles that was obtained by TGA.

The magnetic properties of the PAAMA-coated ultrasmall Gd_2_O_3_ nanoparticles were characterized by recording the M−H curve (−2.0 T ≤ H ≤ 2.0 T) at 300K. The measured M value of the sample was mass-corrected using the net mass (the mass of the Gd_2_O_3_ nanoparticles without PAAMA) that was estimated from the TGA curve. As shown in Figure 8, the nanoparticles are paramagnetic (no hysteresis, zero coercivity, and zero remanence on the M−H curve), similar to the corresponding bulk material [36,37,38]. From the mass-corrected M−H curve (Figure 8), the estimated net M value at 2.0 T was 1.71 emu/g (Table 1). This value at 300 K was appreciable and originates from S = 7/2 of Gd^3+^ (^7/2^S).

### 3.7. r_1_ and r_2_ Values

**Figure 9:**
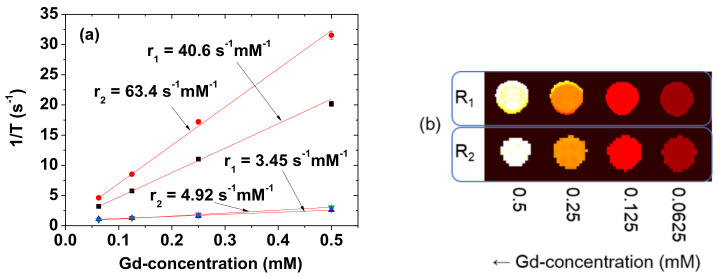
(**a**) Plots of 1/T_1_ and 1/T_2_ of the solution sample and the reference (Dotarem) as a function of the Gd-concentration. The slopes correspond to the r_1_ and r_2_ values, respectively. (**b**) The R_1_ and R_2_ map images showing clear dose-dependent contrast changes.

r_1_ and r_2_ at the 3.0 T MR field were estimated from the slopes of the plots of inverse T_1_ and T_2_ water proton spin relaxation times as a function of the Gd-concentration, respectively (Figure 9a). The estimated r_1_ and r_2_ values are 40.6 and 63.4 s^−1^mM^−1^ (r_2_/r_1_ = 1.56), respectively (Table 1). r_1_ value is approximately 10 times higher than those [2,3] of commercial molecular T_1_ MRI contrast agents. These high r_1_ and r_2_ values indicate that the PAAMA-coated ultrasmall Gd_2_O_3_ nanoparticles could strongly induce T_1_ and T_2_ water proton spin relaxations. These were demonstrated in vitro by measuring the R_1_ and R_2_ map images, in which dose-dependent contrast changes were clearly observed by an increase in the Gd-concentration (Figure 9b). The particle size of the core Gd_2_O_3_ nanoparticles is important for the r_1_ value. The previous study suggested that the optimal particle diameter for the r_1_ value is 1.0 to 2.5 nm [39]. The observed particle diameter is also within this size range.

### 3.8. In Vivo T_1_ MR Images at the 3.0 T MR Field

**Figure 10:**
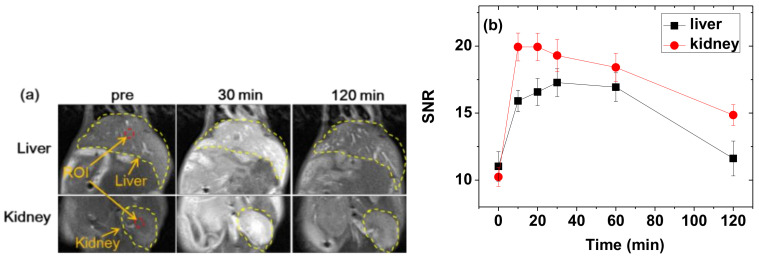
(**a**) In vivo T_1_ MR images of the liver and kidneys before and after the intravenous administration of the aqueous solution sample to the mice tails. Small red dotted circles = ROI and yellow dotted lines = liver or kidney. (**b**) Plots of SNR of ROI as a function of time (*p*-values between 0 timepoint and the other timepoints: *p*-value = 0.029 * for 10, 20, 30, and 60 min for both the liver and kidneys, *p*-value = 0.486 for 120 min for the liver, and *p*-value = 0.032 * for 120 min for the kidneys). “pre” = before administration, “SNR” = signal-to-noise ratio, and “ROI” = region-of-interest.

To demonstrate the effectiveness of the PAAMA-coated ultrasmall Gd_2_O_3_ nanoparticles as a T_1_ MRI contrast agent, in vivo T_1_ MR images were obtained at the 3.0 T MR field before and after the administration of an aqueous solution sample into the mice tail veins. As shown in Figure 10a, positive-contrast enhancements were clearly observed in the liver and kidneys after the administration, thus confirming that the nanoparticles functioned as a T_1_ MRI contrast agent. The signal-to-noise ratios (SNR) of regions-of-interest (ROI) (labeled as dotted circles in Figure 10a) were plotted as a function time (Figure 10b) and indicated that the SNRs reached the maxima ~30 min after the administration before decreasing with time because of the excretion of the nanoparticles from the liver and kidneys. These excretions were due to the ultrasmall particle sizes of the nanoparticles, as observed in various ultrasmall nanoparticle systems [10,11,12]. The T_1_ MR images and SNR plots indicate a fast requisition of the nanoparticles by macrophages, so the trend of these nanoparticles is to be quickly accumulated in the kidneys and liver. The average core particle diameter of the nanoparticles is 1.8 nm. Thus, the surface-coating with PAAMA mainly contributed to the observed large hydrodynamic diameter (a_avg_ = 9.0 nm). However, it is known that polymeric nanoparticles with large hydrodynamic diameters (>100 nm) [40] or large molecular weights (~16.2 kD) [41] could be excreted through the renal system. Therefore, the observed renal excretion might be because most part of the nanoparticles are polymers. In addition, the mice survived after in vivo MRI experiments, thereby confirming the good biocompatibility of the synthesized nanoparticles. These results further indicate that the PAAMA-coated ultrasmall Gd_2_O_3_ nanoparticles are a potential T_1_ MRI contrast agent.

## 4. Discussion

**Figure 11:**
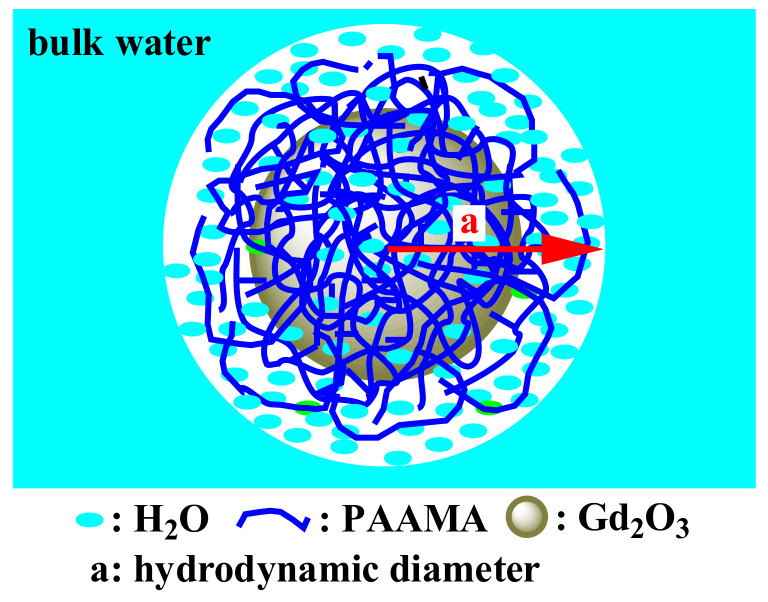
Model picture showing the numerous water molecules that were attracted by the PAAMA polymers coating the ultrasmall Gd_2_O_3_ nanoparticle surface, which availed a large hydrodynamic diameter (a) and, as a result, a very high r_1_ value and good colloidal stability.

The obtained high r_1_ value of the PAAMA-coated ultrasmall Gd_2_O_3_ nanoparticles is attributable to the hydrophilic PAAMA coating on the nanoparticle surface in addition to the ultrasmall core particle size. Owing to the presence of ~288 COO^−^ groups per nanoparticle, as previously mentioned, the PAAMA-coated ultrasmall Gd_2_O_3_ nanoparticles could strongly attract numerous water molecules to the nanoparticle surface, which eases the diffusion of the water molecules around the nanoparticle, thereby causing heavy hydration (a large hydrodynamic diameter) (Figure 11). Therefore, many water molecules interacted with many Gd^3+^ on the nanoparticle surface, thus achieving an extremely high r_1_ value based on the inner-sphere model [2,3].

Herein, the relevance of PAAMA as a surface-coating ligand is not just limited to the surface-coating of the ultrasmall Gd_2_O_3_ nanoparticles. Each PAAMA possesses ~48 COOH groups. Therefore, functional molecules, such as drugs and cancer-targeting ligands, could be easily attached to the PAAMA-coated ultrasmall Gd_2_O_3_ nanoparticles by conjugating them to the free COOH groups of PAAMA through the amide bond, making the nanoparticles relevant cancer theragnostic agents. In addition, other imaging agents such as dyes would be conjugated to the nanoparticles to make the nanoparticles suitable multimodal imaging agents.

Compared with other hydrophilic biocompatible polymers with many carboxylic groups, such as poly(acrylic acid) (PAA) [42] and poly(methyl vinyl ether-alt-maleic acid) (PMVEMA) [43], their average hydrodynamic diameters are generally large, and all r_1_ values are approximately 10 times higher than those [2,3] of commercial molecular contrast agents (Table 2). These results are similar to those reported herein and indicate that, as explained above, such polymers allow heavy hydration of numerous water molecules around the nanoparticle surface. Therefore, they afford extremely high r_1_ values and good colloidal stability. Hence, hydrophilic biocompatible polymers comprising many carboxylic groups are good candidates as surface-coating ligands of ultrasmall Gd_2_O_3_ nanoparticles to be employed as T_1_ MRI contrast agents. Further, such hydrophilic polymer coatings impart the nanoparticles with good biocompatibility, as confirmed by the very low cellular toxicities in this (Figure 7) and previous works [42,43].

## 5. Conclusions

PAAMA was used as a surface-coating ligand of ultrasmall Gd_2_O_3_ nanoparticles (d_avg_ = 1.8 nm); the results are summarized below.

(1) The abundant carboxylic groups in PAAMA allowed its strong bonding to the nanoparticle surface and the attraction of numerous water molecules around the nanoparticle surface, thereby achieving a large a_avg_ of 9.0 nm. This led to an extremely high r_1_ value of 40.6 s^−1^mM^−1^ (r_2_/r_1_ = 1.56) and good colloidal stability, as confirmed by the high zeta potential and absence of precipitation of the nanoparticles in the aqueous solution;
(2) The biocompatible PAAMA coating resulted in extremely low cellular toxicity;
(3) The observed r_1_ value was ~10 times higher than those of commercial molecular contrast agents. Thus, strong positive-contrast enhancements in the in vivo T_1_ MR images were observed. However, more studies, including the pharmacokinetic study, are required to further demonstrate the possibility of using the synthesized nanoparticles as a powerful T_1_ MRI contrast agent.

## Figures and Tables

**Table 1:** Summary of the PAAMA-coated ultrasmall Gd_2_O_3_ nanoparticles.

d_avg_ (nm)	a_avg_ (nm)	ξ_avg_ (mV)	Average Surface-Coating Amount			Net M at 2.0 T and 300 K (emu/g) ^4^	Water Proton Spin Relaxivity at 3.0 T and 22 °C (s^−1^ mM^−1^)	
			P ^1^ (wt.%)	σ ^2^ (nm^−2^)	N ^3^		r_1_	r_2_
1.8 ± 0.1	9.0 ± 0.2	−43.9 ± 0.2	40.3 ± 0.2	0.48 ± 0.05	6 ± 1	1.71 ± 0.05	40.6 ± 0.1	63.4 ± 0.1

^1^ Average surface-coating amount (in wt.%) of the PAAMA polymers per nanoparticle. ^2^ Average grafting (or coating) density (average number of polymers coating a nanoparticle unit surface area). ^3^ Average number of PAAMA polymers coating a nanoparticle. ^4^ Mass-corrected net M value of ultrasmall Gd_2_O_3_ nanoparticles without PAAMA.

**Table 2:** r_1_ and r_2_ values of various hydrophilic biocompatible polymer-coated ultrasmall Gd_2_O_3_ nanoparticles and a commercial molecular agent (for reference).

Chemical	Polymer	MW (amu)	d_avg_ (nm)	a_avg_ (nm)	Water Proton Spin Relaxivity at 3.0 T and 22 °C (s^−1^mM^−1^)		Ref.
					r_1_	r_2_	
**Gd_2_O_3_**	**PAAMA**	**3000**	**1.8**	**9.0**	40.6	63.4	This work
Gd_2_O_3_	PAA ^1^	5000	2.0	6.3	31.0	37.4	[42]
Gd_2_O_3_	PMVEMA ^2^	80,000	1.9	19.8	36.2	74.0	[43]
Gd-DOTA ^3^	-	-	-	-	3.45	4.92	This work

^1^ Poly(acrylic acid). ^2^ Poly(methyl vinyl ether-alt-maleic acid). ^3^ Commercial Gd-chelate contrast agent: Dotarem^®^ (meglumine gadoterate) (Guerbet, France).
